# Lateral femoral traction pin entry: risk to the femoral artery and other medial neurovascular structures

**DOI:** 10.1186/1749-799X-5-4

**Published:** 2010-01-22

**Authors:** John Y Kwon, Catherine E Johnson, Paul Appleton, Edward K Rodriguez

**Affiliations:** 1 Harvard Combined Orthopaedic Residency Program, Department of Orthopaedic Surgery, Massachusetts General Hospital, 55 Fruit Street, Boston, MA 02114, USA; 2 Department of Orthopaedic Surgery, Orthopaedic Trauma, Beth Israel-Deaconess Medical Center, 330 Brookline Avenue, Boston, MA 02215-5491, USA

## Abstract

**Background:**

Femoral skeletal traction assists in the reduction and transient stabilization of pelvic, acetabular, hip, and femoral fractures when splinting is ineffective. Traditional teaching has recommended a medial entry site for insertion of the traction pin in order to minimize injury to the femoral artery as it passes through Hunter's canal. The present anatomical study evaluates the risk to the femoral artery and other medial neurovascular structures using a lateral entry approach.

**Methods:**

Six embalmed cadavers (twelve femurs) were obtained for dissection. Steinman pins were drilled from lateral to medial at the level of the superior pole of the patella, at 2 cm, and at 4 cm proximal to this point. Medial superficial dissection was then performed to identify the saphenous nerve, the superior medial geniculate artery, the adductor hiatus, the tendinous insertion of the adductor magnus and the femoral artery. Measurements localizing these anatomic structures relative to the pins were obtained.

**Results:**

The femoral artery was relatively safe and was no closer than 29.6 mm (mean) from any of the three Steinman pins. The superior medial geniculate artery was the medial structure at most risk.

**Conclusions:**

Lateral femoral traction pin entry is a safe procedure with minimal risk to the saphenous nerve and femoral artery. Of the structures examined, only the superior medial geniculate artery is at a risk of iatrogenic injury due to its position. The incidence of such injury in clinical practice and its clinical significance is not known. Lateral insertion facilitates traction pin placement since it minimizes the need to move the contralateral extremity out of the way of the drilling equipment or the need to elevate or externally rotate the injured extremity relative to the contralateral extremity.

## Background

Skeletal traction via a femoral or tibial traction pin assists in the reduction and transient stabilization of acetabular fractures with or without concomitant hip dislocation, pelvic vertical shear injuries, foreshortened femoral shaft fractures, and other pelvic, hip or femur injuries where splinting is not effective. Placement of a femoral or a tibial traction pin involves the risk of ligamentous knee injury, intramedullary canal contamination, vascular and/or nerve injury, intra-articular contamination, and generation of a stress riser [1,2]. Traditional teaching has recommended a medial entry site with blunt dissection for insertion of the traction pin to minimize risk of injury to the femoral artery as it passes through Hunter's canal [3]. However, a review of the literature reveals no anatomic justification for this practice. In addition, medial entry for traction pin placement can be technically more demanding as the contralateral extremity often blocks drill positioning. This often requires manipulation of the injured extremity to either elevate it relative to the contralateral extremity or to externally rotate it. Alternatively the contralateral extremity needs to be moved out of the way. The objectives of this anatomical study were to evaluate the risk to the femoral artery and other medial neurovascular structures using a lateral pin entry approach, and to evaluate the optimal position for lateral entry traction pin placement.

## Methods

**Figure 1:**
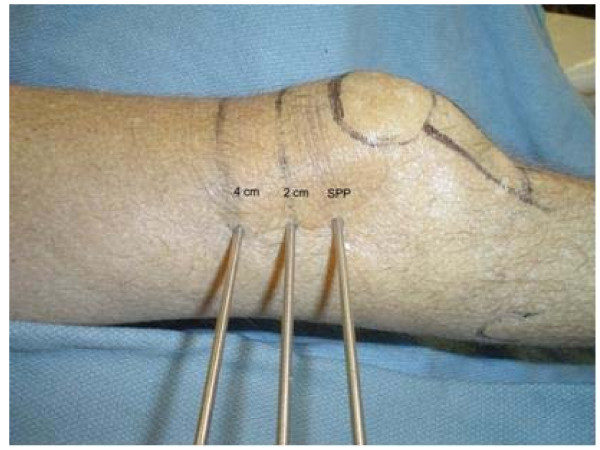
**Photograph showing 4 mm Steinman pins inserted at the distal femur from lateral to medial at the level of the superior pole of the patella (SPP), at 2 cm, and at 4 cm proximal to SPP**.

Six embalmed cadavers (twelve femurs) were obtained for dissection. For each leg, the superior pole of the patella (SPP) was palpated and the skin marked with a transverse line. Similar marks were made at 2 cm and 4 cm proximal to the SPP. The knee was held in full extension with neutral extremity rotation. The distal femur was palpated laterally for the midline position of the femur in the anterior/posterior plane. A small skin incision was made at the midline and a 4 mm Steinman pin was drilled from lateral to medial exiting the medial skin. This was repeated at the 2 cm and 4 cm marks with additional pins (Figure 1).

**Figure 2:**
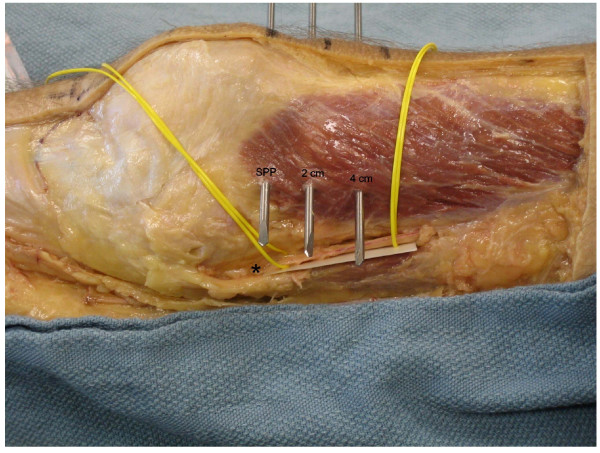
**Cadaveric dissection of the medial knee showing the anatomic location of the saphenous nerve (asterisk) in relation to the 3 Steinman pins**.

**Figure 3:**
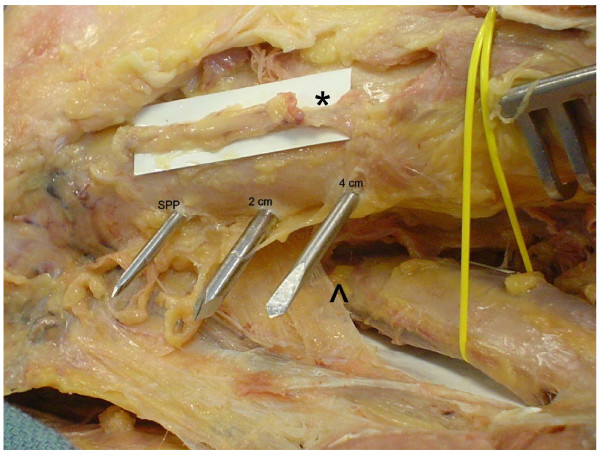
**Cadaveric dissection of the medial knee showing the anatomic location of the superior medial geniculate artery (asterisk) and femoral artery passing through the adductor hiatus (arrow head) in relation to the 3 Steinman pins**.

Medial superficial dissection was then performed and the saphenous nerve was identified (Figure 2). Measurements of the direct anterior to posterior distance from each of the 3 Steinman pins to the saphenous nerve were obtained. Further dissection was then performed and the superior medial geniculate artery was identified (Figure 3). Similar measurements were obtained from each of the 3 pins. Additional dissection was performed to verify that the pins exited from the mid femur in the anterior posterior plane and did not skive anterior or posteriorly.

**Figure 4:**
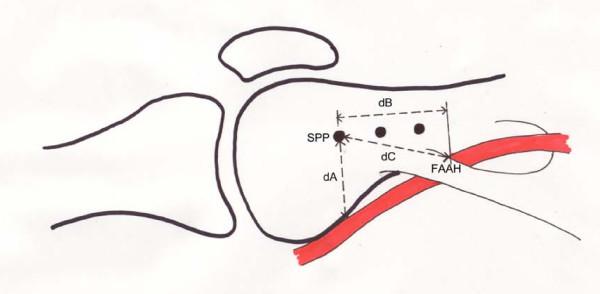
**Schematic drawing of distances characterizing the position of the femoral artery relative to the pins**. Distance (dB) is from superior pole of patella (SPP) to femoral artery crossing the adductor hiatus (FAAH). Distance (dC) is diagonally from each Steinman pin to the FAAH. Distance (dA) is posteriorly from each Steinman pin to the femoral artery.

The adductor hiatus, the tendinous insertion of the adductor magnus and the femoral artery were then identified. The area at which the femoral artery crossed the adductor hiatus (FAAH) was visualized in each case. Measurements characterizing this anatomic landmark relative to the pins were obtained. These include the distance (dB) from a line drawn from the SPP Steinman pin to an anterior to posterior line extending from the FAAH, the diagonal distance (dC) from each Steinman pin to the FAAH, and the anterior-posterior distance (dA) from each Steinman pin to the femoral artery (either proximal or distal to the point where the artery crosses the adductor hiatus) (Figure 4).

The small amount of cadavers precludes a statistical analysis or anatomical differences.

## Results

Six cadavers were dissected for a total of 12 femurs. There were 5 male cadavers and 1 female cadaver. Cadaver #4 was found to have a right total knee arthroplasty and the superior medial geniculate artery was unidentifiable. All other neurovascular structures were identified in the remaining cadavers.

The mean distance (dB) from the superior pole of the patella SPP to the anterior to posterior line extending from the FAAH was 55.5 mm.

The mean anterior to posterior distances from the SPP pin, the 2 cm pin, and the 4 cm pin to the saphenous nerve were 36.8 mm, 35.2 mm and 33.8 mm respectively.

The mean anterior to posterior distances from the SPP pin, the 2 cm pin, and the 4 cm pin to the superior medial geniculate artery were 9.4 mm, 11.5 mm and 12.9 mm respectively.

The mean diagonal distances (dC) from the SPP pin, the 2 cm pin, and the 4 cm pin to the FAAH diagonally were 59.8 mm, 44.5 mm and 33.9 mm respectively.

The mean anterior to posterior distance (dA) from the SPP pin, the 2 cm pin, and the 4 cm pin to the femoral artery were 35.8 mm, 31.3 mm and 29.6 mm respectively.

## Discussion

Femoral skeletal traction has been used for over a century with advent and wide spread use during the World Wars. The initial use of tongs was improved by Fritz Steinmann in 1907 who advocated the use of two pins driven into the femoral condyles [4].

Traditional teaching has recommended a medial entry site with blunt dissection for insertion of the traction pin due to concerns of iatrogenic injury to the femoral artery as it passes through Hunter's canal. Although a widely accepted technique, there has been no prior anatomic study to justify this practice. This study, to our knowledge, is the first and only study reported in the literature addressing this.

The femoral artery is relatively safe when pin placement is performed from a lateral entry point. Instead, the superior medial geniculate artery is the medial neurovascular structure at most risk with lateral pin entry. The average distances from a pin placed at the superior patella pole to the superior medial geniculate artery were 9.4 mm, 11.5 mm from 2 cm proximal, and 12.9 mm from 4 cm proximal. In contrast, the average distances from a pin placed at the superior patella pole to the saphenous nerve were 36.8 mm, 35.2 mm from 2 cm proximal, and 33.8 mm from 4 cm proximal. Similarly, the femoral artery had a relatively wide safe zone with the average distances from a pin placed at the superior patella pole to the femoral artery being 35.8 mm, 31.3 mm from 2 cm proximal, and 29.6 mm from 4 cm proximal.

We propose that lateral pin entry for femoral traction pins is safe. The superior medial geniculate artery is the structure at most risk of injury, particularly when pin placement is done at the level of the superior patella pole. The safe distance from the superior medial geniculate artery was increased as the pin was placed more proximally at 2 cm and 4 cm from the superior patella pole. While a more proximal pin position could be advocated to protect the superior medial geniculate artery, with the safest location for the entry point being 4 cm proximal to the superior patella pole, this may result in a stress riser through the meta-diaphyseal area of the femur once the pin is removed. Also, the safe zone for the femoral artery decreases with more proximal pin placement. We therefore advocate that pin placement be made 2 cm proximal to the superior patellar pole when performing a lateral approach. This will increase the margin of safety for the superior medial geniculate artery while preserving a generous safe zone for the saphenous nerve and the femoral artery.

The incidence of injury to the superior medial geniculate artery and its clinical significance during traditional femoral traction pin placement is unknown. Ashok Reddy, et al. demonstrated in a cadaveric study that the medial femoral condyle is supplied primary by the superior medial geniculate artery and other lesser branches from the popliteal artery [5]. While the lateral femoral condyle enjoys a rich intraosseous supply, the intraosseous supply to the medial femoral condyle appeared to consist of a single nutrient vessel supplying the subchondral bone with an apparent watershed area of limited supply. Theoretically iatrogenic injury during traction pin placement could result in avascular necrosis. Whether lateral versus medial pin placement offers a decreased risk to the geniculate artery and the clinical sequela is unknown.

The authors recognize potential weaknesses in this study. Our sample size was relatively small, 5 out of 6 cadavers were male, and race was unknown. Inherent to any cadaveric anatomic study is the applicability of the data collected across age, sex and race. However, the anatomic relationships of the various neurovascular structures studied were consistent enough in the 12 knees dissected that the findings are still useful and may apply to a larger study population.

Another arguable weakness is the lack of lateral dissection. However, this study does not purport to identify all structures at risk during transcutaneous osseous insertion of distal femoral traction pins nor to be an anatomic study of the distal femur. Injury to the femoral artery and the potential for devastating vascular injury is the true rational for traditional medial entry and challenging this long-held belief was the purpose of our work. Current practice of medial entry offers protection to the femoral artery by percutaneous blunt dissection down to bone medially before inserting the pin disregarding the safety of the lateral neurovascular structures as pins come out blindly through the lateral side. In our study we offer the same protection to any lateral structures that may be at risk when implementing our lateral insertion technique by using the same technique.

## Conclusions

Although our results suggest that lateral pin entry is safe, one must still use caution. Proper sterile technique is essential as is careful blunt dissection of soft tissues prior to pin entry. The femoral midpoint in the anterior-posterior plane should be identified atraumatically as vigorous pin movement can endanger the lateral vascular structures. The pins should be drilled level with the extremity in neutral alignment as pins directed in a medial inferior direction may in fact endanger the femoral artery.

## Competing interests

The authors declare that they have no competing interests.

## Authors' contributions

JK: performed dissections and data collection and analysis, writing of manuscript.

CJ: performed dissections and data collection and analysis.

EKR: intellectual contribution, edited manuscript.

PA: intellectual contribution, edited manuscript.
